# *In vitro* chondrogenic potency of surplus chondrocytes from autologous transplantation procedures does not predict short-term clinical outcomes

**DOI:** 10.1186/s12891-018-2380-4

**Published:** 2019-01-10

**Authors:** Ashraful Islam, Vegard Fossum, Ann Kristin Hansen, Ilona Urbarova, Gunnar Knutsen, Inigo Martinez-Zubiaurre

**Affiliations:** 1 Department of Clinical Medicine, UiT The Arctic University of Norway, Tromsø, Norway; 2 Department of Orthopaedic Surgery, University Hospital of Northern Norway, Tromsø, Norway; 3 Department of Medical Biology, Tromsø University Proteomics Platform, UiT The Arctic University of Norway, Tromsø, Norway

**Keywords:** ACI, Biomarkers, In vitro chondrogenesis, Clinical outcome, Proteomics, P4HA1, CD166, Cartilage, 3D culture, Chondrocytes

## Abstract

**Background:**

Autologous chondrocyte implantation (ACI) has been used over the last two decades to treat focal cartilage lesions aiming to delay or prevent the onset of osteoarthritis; however, some patients do not respond adequately to the procedure. A number of biomarkers that can forecast the clinical potency of the cells have been proposed, but evidence for the relationship between in vitro chondrogenic potential and clinical outcomes is missing. In this study, we explored if the ability of cells to make cartilage in vitro correlates with ACI clinical outcomes. Additionally, we evaluated previously proposed chondrogenic biomarkers and searched for new biomarkers in the chondrocyte proteome capable of predicting clinical success or failure after ACI.

**Methods:**

The chondrogenic capacity of chondrocytes derived from 14 different donors was defined based on proteoglycans staining and visual histological grading of tissues generated using the pellet culture system. A Lysholm score of 65 two years post-ACI was used as a cut-off to categorise “success” and “failure” clinical groups. A set of predefined biomarkers were investigated in the chondrogenic and clinical outcomes groups using flow cytometry and qPCR. High-throughput proteomics of cell lysates was used to search for putative biomarkers to predict chondrogenesis and clinical outcomes.

**Results:**

Visual histological grading of pellets categorised donors into “high” and “low” chondrogenic groups. Direct comparison between donor-matched in vitro chondrogenic potential and clinical outcomes revealed no significant associations. Comparative analyses of selected biomarkers revealed that expression of CD106 and TGF-β-receptor-3 was enhanced in the low chondrogenic group, while expression of integrin-α1 and integrin-β1 was significantly upregulated in the high chondrogenic group. Additionally, increased surface expression of CD166 was observed in the clinical success group, while the gene expression of cartilage oligomeric matrix protein was downregulated. High throughput proteomics revealed no differentially expressed proteins from success and failure clinical groups, whereas seven proteins including prolyl-4-hydroxylase 1 were differentially expressed when comparing chondrogenic groups.

**Conclusion:**

In our limited material, we found no correlation between in vitro cartilage-forming capacity and clinical outcomes, and argue on the limitations of using the chondrogenic potential of cells or markers for chondrogenesis as predictors of clinical outcomes.

**Electronic supplementary material:**

The online version of this article (10.1186/s12891-018-2380-4) contains supplementary material, which is available to authorized users.

## Background

Articular cartilage injuries may develop into osteoarthritis (OA) [1]. However, the management of cartilage lesions in the synovial joints still represents a weighty clinical challenge. Since the mid 90’s, autologous chondrocyte implantation (ACI) has been available as a method to ameliorate these impairing localised cartilage defects [2]. Successful clinical outcomes of ACI have been reported for up to 20 years [3, 4]. The original technique has experienced refinements such as the introduction of collagen membranes to replace periosteum to cover the defect, the use of characterised chondrocytes to improve the quality of the repair tissue or the more recently matrix-assisted chondrocyte implantation (MACI) where the chondrocytes are seeded in a collagen matrix before implantation [5, 6]. The long-term failure rate of the first generation procedure is in the range between 20 and 40% after 15 years [7, 8], while five-year failure rate of MACI is reported to be 11% [9], mind that the definition of failure is not directly comparable between studies.

To improve the decision-making process around the choice of treatment for patients with localised cartilage defects, it would be of great advantage to have a tool to identify those likely to obtain an optimal outcome of the procedure. Some patient characteristics have been identified, and although the reports are not unanimous, most agree on patient age, preoperative function scores, previous surgeries to the index knee and defect location and age being linked to the surgical outcome [10–12]. Further stratification methods have been pursued by trying to identify biomarkers linked to clinical outcomes from liquid biopsies. Wright et al. reported that increased levels of CD14 and ADAMTS-4 in the preoperative synovial fluid was linked to the poor outcome of the ACI [13]. Some few other studies have assessed synovial fluid or serum for biomarkers of cartilage injury treatment from which limited putative predictive biomarkers have been identified [14, 15]. Additionally, molecular biomarkers to predict treatment outcomes have been explored from the cell sources used in the procedures. Thus, markers found in monolayer cultures such as collagen type II A1 (COL2A1), aggrecan (ACAN), fibroblast growth factor receptor 3 (FGFR-3) and bone morphogenic protein 2 (BMP-2) have been associated with cartilage formation in vivo in a murine model [16]. Stenberg et al. performed a global microarray analysis of surplus cells from ACI and found no links between clinical outcomes and genes linked to cartilage formation in vivo [17].

In the past, it has been demonstrated that even after applying identical isolation and culture conditions, human chondrocytes from different individuals display strikingly different in vitro chondrogenic capacity [18, 19]. Based on such findings, researchers have tried to search for markers that forecast cell chondrogenicity from in vitro expanded cells, in order to recognise the quality of the cells from donors and possibly to improve the quality of the generated tissue [20–23]. However, evidence to support the relationship between the in vitro chondrogenic potency of cells before the implantation and clinical outcomes is lacking. Therefore, it is uncertain whether markers of intrinsic chondrogenic potency could be used as prognostic and quality measures in clinical practice.

In this study, we have explored first if the in vitro chondrogenic potency of leftover cells from ACIs established in pellet cultures could be used as a convenient and reproducible functional bioassay to predict clinical outcomes. Secondly, we evaluated if previously reported markers have predictive clinical or chondrogenic value in our material. Finally, we investigated whole cell lysates by quantitative high-throughput proteomics to identify yet unknown molecular biomarkers that can predict chondrogenesis and clinical outcomes.

## Methods

### Human materials and cell isolation

Chondrocytes were surplus cells from 14 patients treated with autologous chondrocyte implantation and were acquired after the written consent of the patients and approval from the regional ethics committee (REK Nord 2014/920). The isolation protocol has been described previously [24]. Briefly, the ~ 200 mg cartilage specimens were kept in 0.9% NaCl for maximum 2 h before mincing to ~ 1 mm^3^ pieces and enzymatic digestion for 3–4 h in DMEM/HAM’s F-12 (Cat. no. T 481–50, BioChrom Labs) containing collagenase XI (Cat. no. C-9407, Sigma-Aldrich) at a final concentration of 1.25 mg/mL. Chondrocytes released from matrix were serially expanded in DMEM/HAM’s F-12 supplemented with 10% human autologous serum until implantation at passage 3. Surplus cells were cryopreserved in DMEM/HAM’s F-12, 20% foetal bovine serum (FBS; Cat. no. S0115; Biochrom) and 10% dimethyl sulfoxide (DMSO; Cat. no. D2650, Sigma-Aldrich) until inclusion in the study. After careful thawing, the chondrocytes were propagated in high glucose Dulbecco’s Modified Eagle Medium (DMEM; Cat. no. D5796; Sigma-Aldrich) supplemented with L-ascorbic acid (62 mg/L) (Cat. no.103033E; BDH Laboratory), penicillin and streptomycin (1%) (P/S; Cat. no. P4333; Sigma-Aldrich) and 10% FBS at 37 °C in humidified atmosphere containing 5% CO2. The medium was changed twice a week and passaged upon reaching 70–80% confluency.

### Chondrogenesis and 3D cultures

Chondrogenic potential of dedifferentiated chondrocytes was achieved by using both hanging-drop and pellet culture method. For pellet cultures, ex vivo expanded chondrocytes were harvested and prepared at a final concentration of 5 × 10^4^ cells/150 μL per pellet as previously described [25]. Briefly, 5 × 10^4^ cells/well were placed in poly-HEMA (Cat. no. P3932; Sigma-Aldrich) coated conical-bottom 96 well culture plate (Cat. no. 249935; Thermo Scientific) and centrifuged at 1100 g for 10 min to form cell aggregates. For hanging-drops, chondrocytes were dispensed as a 40 μL drop containing 2 × 10^4^ cells/drop on the lid of a Petri dish. Aggregates were formed by gravitational forces as the drop was hanging upside down. After 48 h, spheroids from conical-bottom plates or hanging-drops were collected and cultured on a 24 well ultra-low attachment cell culture plate (Cat. no. 3473; Corning) in a serum-free chondrogenic medium for 21 d at low oxygen (3% O_2_). The chondrogenic medium contained high glucose DMEM, L-ascorbic acid (62 mg/L), P/S (1%), dexamethasone (1 μg/mL) (Cat. no. PZN-3103491; Galenpharma), insulin-transferrin-selenium supplement (ITS) (1:1000) (Cat. no. 354351; BD Biosciences), transforming growth factor β1 (10 ng/mL) (TGF-β1; Cat. no. 100-21C; Peprotech) and BMP-2 (100 ng/mL) (Cat. no. 120-02C; Peprotech). Half of the chondrogenic medium was replaced with fresh chondrogenic medium twice a week.

### Flow cytometry

Monolayer cultured chondrocytes were harvested and prepared at passage 3–4 for surface marker expression by flow cytometry as previously described [25]. Briefly, chondrocytes were harvested and washed three times with cold stain buffer (Cat. no. 554656; BD Biosciences), filtered through a 70 μm cell strainer and prepared on ice as single-cell suspensions to a final concentration of < 1 × 10^6^ cells/100 μL and incubated with antibodies at 1:10 dilution for 1 h. Fluorochrome-conjugated antibodies targeting CD44 (Cat. no. 555479), CD106 (Cat. no. 561679), CD146 (Cat. no. 561013), CD166 (Cat. no. 560903), CD271 (Cat. no. 560927), isotype control PE Mouse IgG2b (Cat. no. 555743) and isotype control PE Mouse IgG1 (Cat. no. 555749) were purchased from BD Biosciences, USA. Samples were analysed using a BD FACSAria III flow cytometer and FlowJo software (Tree Star Inc., USA). Data from three donors were presented as the average of median fluorescence intensity (MFI) +/− standard error.

### Alcian blue staining and Bern score

Metachromatic staining of proteoglycans by Alcian blue was done as previously described [25]. Spheroids from pellet cultures (*n* = 14, diameter ≈ 1 mm) and hanging-drops (*n* = 4, diameter ≈ 0.5 mm) were harvested at day 21, washed in DPBS and fixed in 4% formalin overnight. Fixed spheroids were embedded in 1% agarose and transferred into a paraffin block. Paraffin-embedded sections (4 μm) were dewaxed and stained with Alcian blue solution (Cat. no. A5268; Sigma-Aldrich) for 30 min. Sections were washed for 2 min in distilled water and counterstained with a Nuclear fast red solution (Cat. no. N3020; Sigma-Aldrich) for 5 min. Finally, the sections were washed and dehydrated by a series of ethanol and xylene wash, before mounting a coverslip with Histokit (Cat. no. 1025/500; Glaswarenfabrik Karl Hect). Sections were imaged by bright field light microscopy (Leica DMI6000B). To quantify the in vitro chondrogenic potential, a visual semi-quantitative scoring of tissue sections (Bern score) was applied independently by three different observers [26]. The chondrogenic potential was classified into two groups according to histological outcomes: “Group A” with high chondrogenic potential (Bern score 6–9) and “Group B” with low chondrogenic potential (Bern score < 6) (Table 1).

**Table 1:** Donor characteristics and donor-specific chondrogenic potential of culture expanded chondrocytes in 3D spheroids

Source	Age-ranges	gender	Passage	Hanging-drop culture	Pellet culture	Bern Score
Group A (Bern Score 6–9)						
Donor 1	24–55	F	4	+	+	8
Donor 2		M	6	+	+	7
Donor 3		M	6	–	+	8
Donor 4		M	3	+	+	7
Donor 5		M	3	–	+	7
Donor 6		M	3	–	+	7
Donor 7		F	3	–	+	6
Donor 8		F	3	+	+	6
Group B (Bern Score < 6)						
Donor 9	19–53	M	5	–	+	5
Donor 10		F	4	–	+	4
Donor 11		F	6	–	+	4
Donor 12		M	3	–	+	3
Donor 13		M	5	–	+	3
Donor 14		M	3	–	+	2

### Clinical outcomes and score

Clinical outcomes are from the “ACI-C versus AMIC” study (Clinicaltrials.gov identifier; NCT01458782) where 41 patients in the age of 18–60 years were included and randomised to either autologous chondrocyte and collagen membrane implantation (ACI-C) or autologous matrix-induced chondrogenesis (AMIC). The patients were included and operated in 2011–2014, the ACI-C procedure was done as previously described [7] and Chondro-Gide® membranes were used to cover the defect both for ACI-C and AMIC [27]. Lysholm score and the knee injury and osteoarthritis outcome score (KOOS) reporting patients’ pain, symptoms and disability were recorded at the preoperative stage, one-year and two-year follow-up and subsequently used to evaluate patients’ clinical outcomes. We used Lysholm score of 65 at two-year follow up as a cut-off to categorise clinically success (> 65) and failure (< 65) as suggested by Knutsen et al. [7]. Furthermore, we evaluated clinical outcomes by minimal clinically important difference (MCID), which confers with an increase of 10 points in the Lysholm score after one year of post-treatment, to categorise clinically success group [28]. Both approaches resulted in identical patient allocation in clinical success and failure groups. Patients’ demographic data, symptoms, history, functional score, clinical findings and pain as indicated on a visual analogue scale (VAS) were recorded. Patients’ demographic characteristics, as well as defect location and size, are summarised in Table 2.

**Table 2:** Clinical outcome of patients after two years of ACI. Lysholm score (65% cutoff) after two years was used to divide patients in success and failure group

Source	Age-ranges	Gender	Defect (cm^2^)	VAS		KOOS		Lysholm	
				Pre	2 yr	Pre	2 yr	Pre	2 yr
Success group (> 65% Lysholm)									
Donor 1	19–55	M	2.25	40	3	43.5	82.7	55	90
Donor 2		M	3	40	12	68.5	82.1	69	90
Donor 3		M	4.6	50	10	71.4	83.9	56	86
Donor 4		M	9.75	62	10	62.5	76.9	52	83
Donor 5		F	5.2	31	34	78	82.7	64	78
Donor 6		F	3.6	51	14	68.3	73.8	59	74
Donor 7		F	6	48	51	58.3	70.8	57	69
Donor 8		M	21.5	17	4	72.6	84.8	50	69
Donor 9		M	2.4	67	35	38.7	71.4	58	68
Failure group (< 65% Lysholm)									
Donor 10	31–52	F	1.82	50	69	32.1	54.8	56	62
Donor 11		M	5	51	37	82.7	52.4	64	56
Donor 12		M	3	30	56	36.3	47.6	56	49
Donor 13		F	3.1	60	73	68.5	47.6	55	47
Donor 14		M	1.2	74	76	44	35.7	41	38

### qPCR

Monolayer chondrocytes were harvested at passage 3–6 at the time of establishment of 3D cultures, and RNA was extracted using the RNeasy Plus Mini Kit (Cat. no. 74134; Qiagen) according to the manufacturer’s procedure including DNase I treatment. The RNA concentration was measured using the NanoDrop 2000, and RNA from 13 donors had sufficient quality to use for selected markers expression by qPCR. Using the qScript cDNA Synthesis Kit (Cat. no. 95047; Quanta Biosciences), 285 ng of each sample was transcribed to cDNA. The qPCR was performed as previously described [29]. The qPCR included 5 μL PrecisionFAST mastermix (Cat. no. Precision-FAST-R; PrimerDesign), 0.5 μL hydrolysis probe (all from Applied Biosystems), 2.5 μL H_2_O and 2 μL cDNA (diluted to 2 ng/μL) and was run in 96-well plates (Cat. no. BW-FAST; PrimerDesign) using the StepOnePlus Real-Time PCR system (Applied Biosystems). Hydrolysis probes are summarised in Table 3. The gene for ribosomal protein L13a (RPL13A) was used as the reference gene, and ΔCq was calculated by subtracting the gene of interest from the reference gene, making higher ΔCq reflecting increased gene expression.

**Table 3:** Hydrolysis probes

ITGA1	Hs00235006_m1
ITGA2	Hs00158127_m1
ITGA3	Hs01076879_m1
ITGA5	Hs01547673_m1
ITGA6	Hs01041011_m1
ITGA10	Hs00174623_m1
ITGAV	Hs00233808_m1
ITGB1	Hs00559595_m1
ITGB3	Hs01001469_m1
ITGB4	Hs00236216_m1
ITGB5	Hs00174435_m1
COL1A1	Hs00164004_m1
COL2A1	Hs00264051_m1
MATN3	Hs00159081_m1
NCAM1	Hs00941830_m1
CD44	Hs01075861_m1
ICAM1	Hs00164932_m1
CDH2	Hs00983056_m1
BMPR1A	Hs01034913_g1
BMPR1B	Hs01010965_m1
BMR2	Hs00176148_m1
TGFBR1	Hs00610320_m1
TGFBR2	Hs00234253_m1
TGFBR3	Hs00234257_m1
RPL13A (reference gene)	Hs04194366_1g

### Protein extraction and LC-MS/MS analysis

Three donors with extreme scores from each of the chondrogenic groups and clinical groups were analysed by LC-MS/MS. Monolayer chondrocytes were harvested at passage 3–4, and whole protein was extracted using the TMTsixplex™ Isobaric Mass Tagging Kit (Cat. no. 90064; Thermo Scientific). Briefly, cells were washed 3 times with DPBS and lysed in buffer containing 1% sodium deoxycholate (Cat. no. D6750; Sigma-Aldrich) and 100 mM triethylammonium bicarbonate (TEAB). Cell lysates were incubated with Pierce™ Universal Nuclease (Cat. no. 88700; Thermo Scientific) at room temperature for 15 min and centrifuged at 16000 g for 10 min at 4 °C. The supernatants were collected, and protein concentration was measured using a DC Protein Assay Kit (Cat. no. 5000116; Bio-Rad). Samples containing 100 μg/tube protein were reduced in 5 mM dithiothreitol (Cat. no. D9779; Sigma-Aldrich) for 30 min at 70 °C and followed by incubation with 375 mM iodoacetamide for 30 min in the dark at room temperature. Samples were precipitated overnight in pre-chilled acetone (Cat. no. 270725; Sigma-Aldrich) at − 20 °C and collected as dry pellet after centrifugation at 8000 g for 10 min at 4 °C. Protein pellets (25 μg) were resuspended in 2 M Urea (Cat. no. U1250; Sigma-Aldrich) with 50 mM TEAB. Proteins were digested for 6 h with 1:100 (*w*/w) lysyl endopeptidase (Cat. no. 125–05061; Wako Chemicals). The samples were further diluted to 1 M Urea and digested overnight by 1:20 (w/w) trypsin (Cat. no. V511A; Promega). Peptides from each sample were labelled with the TMTsixplex™ Isobaric Mass Tagging Kit according to the manufacturer’s protocol.

OMIX C18 tips were used for sample clean-up and concentration. Peptide mixtures containing 0.1% formic acid (Cat. no. 28905; Thermo Scientific) were loaded to a Thermo Fisher Scientific EASY-nLC1000 system and EASY-Spray column (C18, 2 μm, 100 Å, 50 μm, 50 cm). Peptides were fractionated using a 2–100% acetonitrile (Cat. no. 51101; Thermo Scientific) gradient in 0.1% formic acid over 180 min at a flow rate of 250 nL/min. The separated peptides were analysed using a Thermo Scientific Q-Exactive mass spectrometer. Data were collected in a data-dependent mode using a Top10 method. Raw data were processed using MaxQuant (v 1.5.6.0) with the integrated Andromeda search engine. MS/MS data were searched against the UniProt human database from November 2016. A false discovery rate (FDR) of 0.01 was needed to yield a protein identification.

Statistical validation of protein regulation was performed using the Perseus 1.5.6.0 software. All contaminants were filtered out, and intensity values were log2-transformed for subsequent analysis. The log2-transformed intensities were normalised by adjustment. Data were grouped as group “A (high) and B (low)” for chondrogenesis and “success and failure” for clinical outcomes. Data were then analysed with a minimum of two valid values in each group. A t-test visualised as a volcano plot was generated to identify potentially regulated proteins in the chondrogenic and clinical groups by a permutation-based FDR < 0.05.

### Western blots

Three donors from each chondrogenic group were analysed by western blot. The protein input was 35 μg/lane in TruPage gels (Cat. no. PCG2004; Sigma-Aldrich). The protein was separated along with a BLUeye Prestained Protein Ladder (Cat. no. PM007–0500; Sigma-Aldrich) and MagicMark™ XP Western Protein Standard Ladder (Cat. no. LC5602; Novex). Proteins were transferred to a PVDF membrane, blocked for 2 h in PBS-Tween (0.05%) buffer containing BSA (2%) and incubated with 0.1 μg/mL of prolyl 4-hydroxylase 1 antibody (P4HA1; Cat. no. NB100–57852; Novus Biologicals) overnight at 4 °C. The membrane was incubated with secondary donkey anti-goat antibody (Cat. no. HAF109; Novus Biologicals) for 1 h at room temperature. Finally, a chemiluminescence detection solution (Cat. no. 170–5040, BioRad) was applied to the membrane before acquiring the images using an ImageQuant LAS 4000 CCD camera. Beta-actin antibody (Cat. no. AB8227; Abcam) and goat anti-rabbit antibody (Cat. no. AB6721; Abcam) were used as loading control and secondary antibody for beta-actin, respectively. Relative density was assessed using ImageJ software to compare the chondrogenic groups.

### Statistical analysis

The Bern score between the two chondrogenic groups was plotted as dot density and analysed using Mann-Whitney U comparison. Differences in preoperative, one-year and two-year follow-up scores of VAS, Lysholm and KOOS total between two chondrogenic groups were studied using Mann-Whitney U comparison. Differences in gene expression between the chondrogenic groups and clinical groups were analysed using nested linear regression and Benjamini-Hochberg *p*-value adjustment. Pearson correlation (*r*) was performed to investigate the relationship between in vitro chondrogenic potentials and clinical outcomes. The significance level for all tests was set to < 0.05.

## Results

### The donor-specific chondrogenic potential of surplus chondrocytes in 3D cultures

**Fig. 1:**
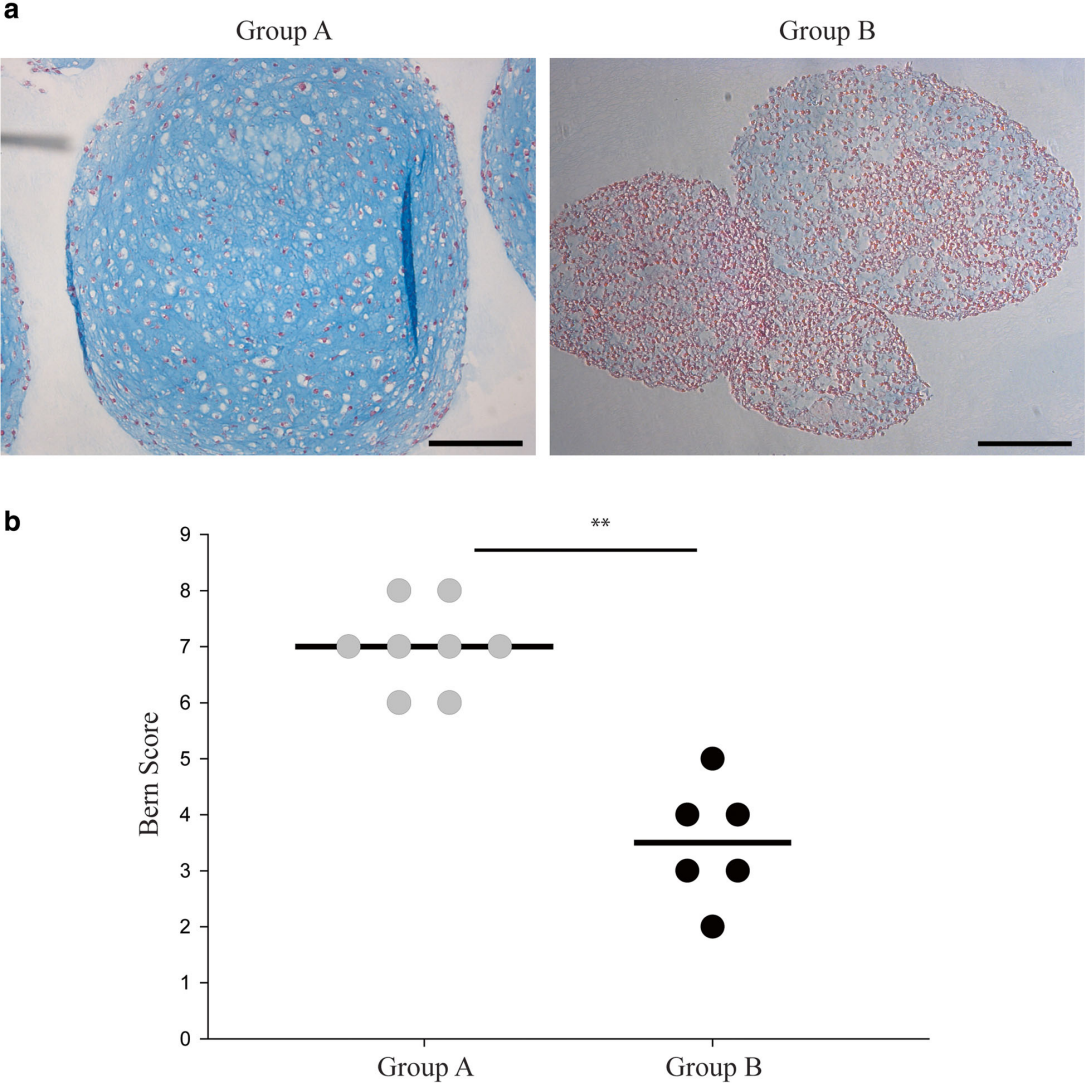
Chondrogenesis of culture-expanded chondrocytes in 3D pellets propagated in chondrogenic medium. **a** Representative bright light microscopy images of histological sections, stained for proteoglycans with Alcian blue and the nuclei counterstained with Sirius red, corresponding to “Group A” and “Group B” with high and low chondrogenic potential, respectively. **b** Semi-quantitative analysis representing the histological scoring of Alcian blue stained 3D pellets demonstrated significant differences between the two groups. Scale bar: 200 μm and significance level, *p* (**) < 0.005

**Fig. 2:**
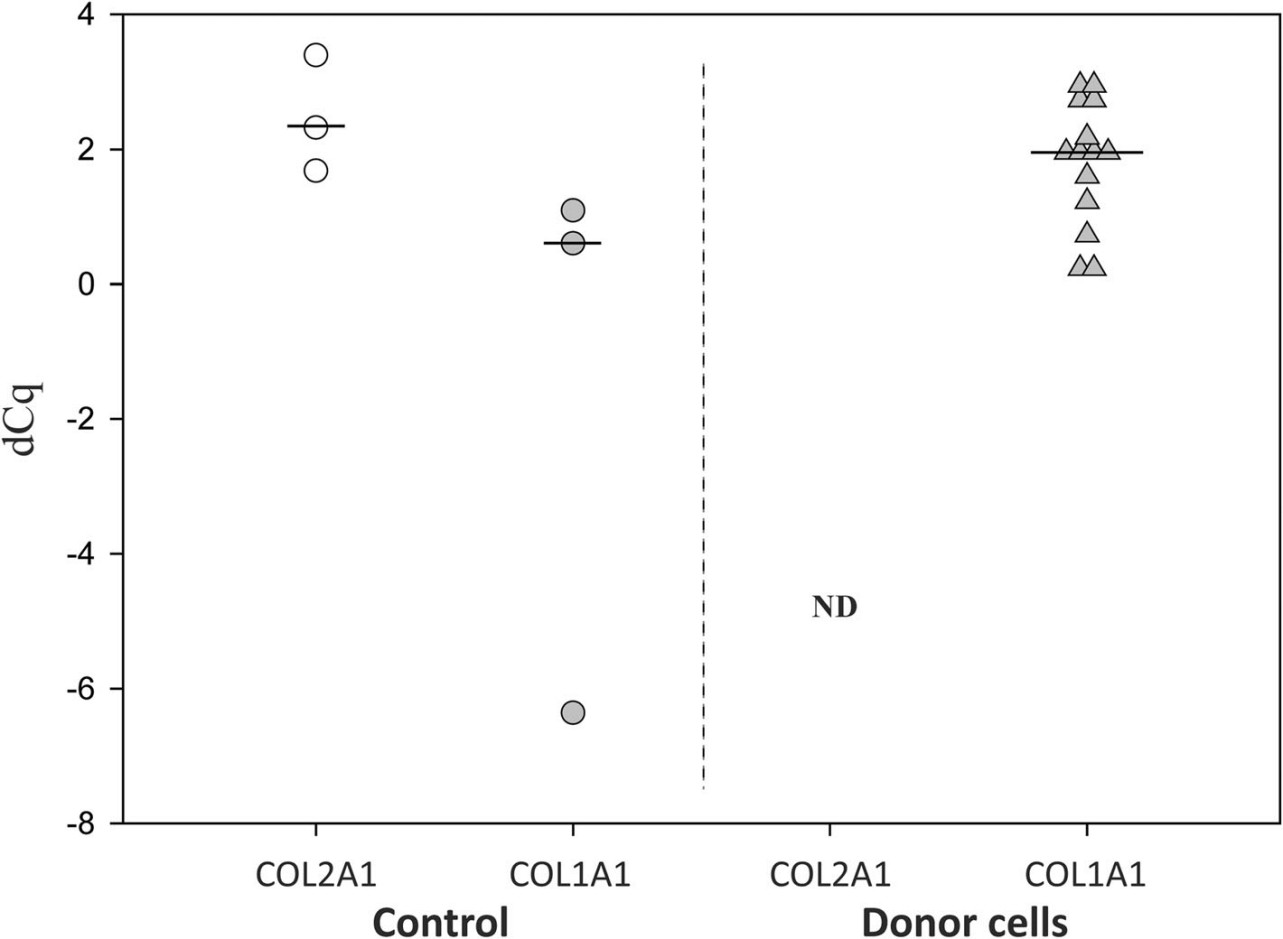
Dedifferentiation of chondrocytes in monolayer culture. Comparison of COL2A1 and COL1A1 gene expression by qPCR in control and donor cells (*n* = 14, passage 3–6) in monolayer culture. Control: Freshly isolated OA chondrocytes; ND: Not detected

Chondrocytes from different donors displayed distinct in vitro chondrogenic potential in 3D cultures (Fig. 1a and Additional file 1: Figure S1). Pellet cultures were achievable with cells from all donors. Semi-quantitative assessments of constructs by visual histological grading system (Bern score) allowed the categorisation of all donors into two groups: “Group A” (8 donors) and “Group B” (6 donors) with high and low chondrogenic characteristics, respectively (Fig. 1b). Hanging-drop cultures were, on the other hand, successful in only half of the donors in group A and none in group B, indicating that the ability of cells to form cartilage-like micro-tissues by hanging-drops had a positive correlation with the intrinsic in vitro chondrogenic potential in pellets (Table 1). To exclude the possible influence of passage number in chondrogenic outcomes, chondrogenesis was evaluated for some donors across passages 3 to 6. Bern score demonstrated no differences in chondrogenic features in constructs made by same donor-cells across different passages. Of importance, investigation of dedifferentiation in 2D culture confirmed that chondrocytes from all donors were fully dedifferentiated as shown by drastic reduction of COL2A1 vs COL1A1 gene expression (Fig. 2). Donor characteristics, summarised in Table 1, showed that the distribution of age, gender and passage is comparable between the two chondrogenic groups.

### In vitro chondrogenic potential do not predict clinical outcomes

**Fig. 3:**
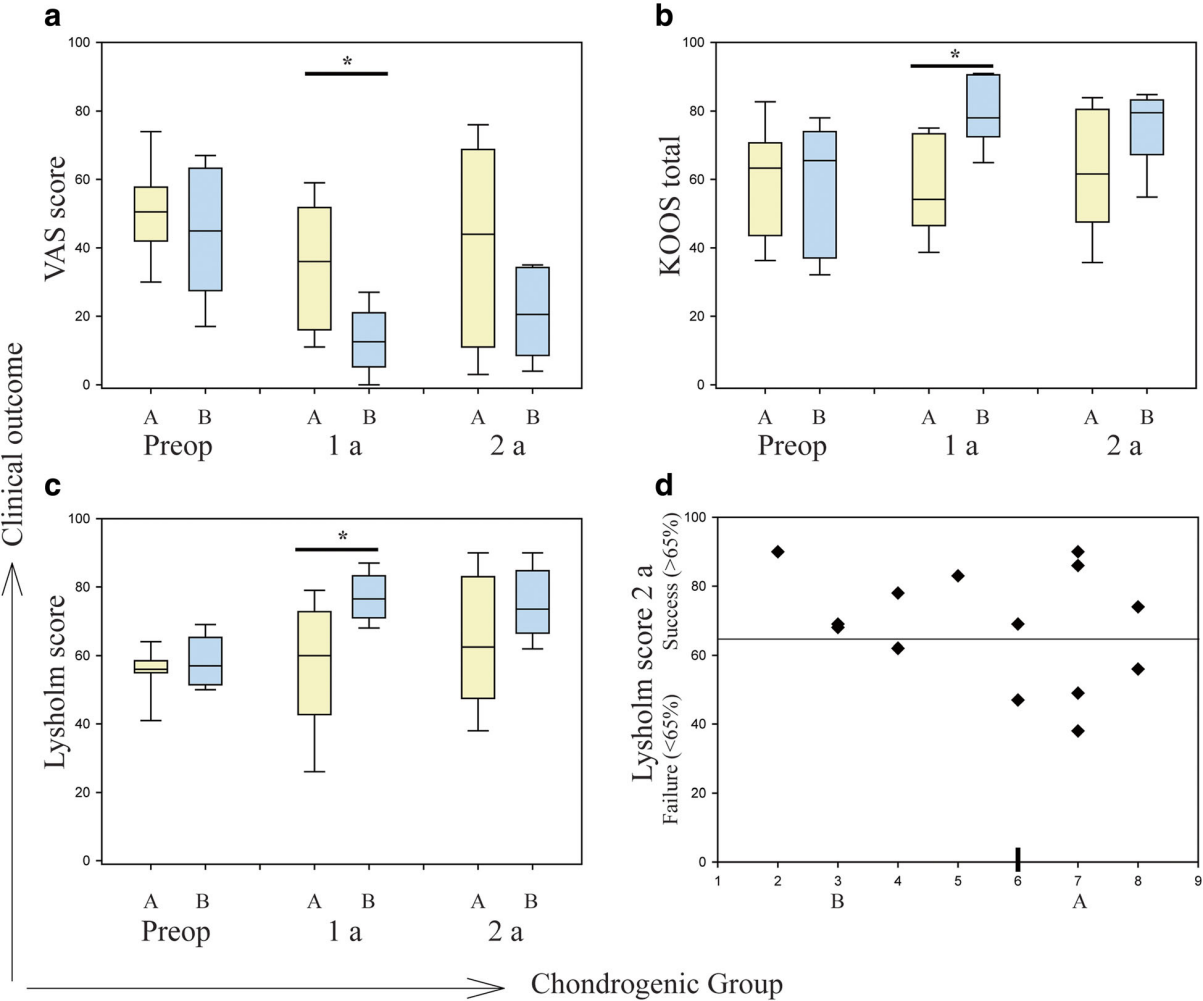
Comparison of donor-matched chondrogenic potential with clinical outcomes. VAS score (**a**), KOOS total (**b**) and Lysholm score (**c**) were plotted against chondrogenic Group A and Group B at the preoperative stage, one-year and two-year follow-up after ACI. **d** Patient distribution using Lysholm score (cut-off < 65) at two-year follow-up demonstrated clinical success and failure groups and their no significant association (*r* = −.308, *p* = 0.284) with in vitro chondrogenic potentials. Significance level, *p* (*) < 0.05

To explore if the in vitro chondrogenic potency of surplus cells from ACIs could be used as a functional bioassay to predict clinical outcomes, we compared VAS, total KOOS and Lysholm score to the chondrogenic groups at baseline, one and two-year after ACI surgery. Patients’ demographic characteristics and defect location and size are summarised in Table 2 along with the clinical outcomes. Preoperatively, the median VAS score for patients in chondrogenic groups A and B was 50.50 (interquartile range (IQR) 15.75) and 45 (IQR: 35.75), respectively, in a scale ranging from 0 to 100, with 100 representing worst imaginable pain. Median VAS score at first-year follow-up for group A and B was 36 (IQR: 35.75) and 12.50 (IQR: 15.75), respectively. At one-year follow-up, significantly reduced VAS score was observed in patients from group B compared to group A. At the two-year follow-up, the median VAS score was 44 and 20.50 in group A (IQR: 57.75) and group B (IQR: 25.75), respectively (Fig. 3a).

Both KOOS total and Lysholm scores range from 0 to 100, with 100 representing unimpaired knee function. The median KOOS total preoperatively was 63.30 (IQR: 27.05) and 65.50 (IQR: 36.90), for patients in chondrogenic groups A and B respectively. At one-year follow-up, the median KOOS total was significantly increased in group B (78, IQR: 18.13) compared to group A (54.15, IQR: 26.80). Median KOOS total at the two-year follow-up was 61.60 and 79.50 for group A and B, respectively (Fig. 3b). In addition, preoperative median Lysholm score was 56 (IQR: 3.50) and 57 (IQR: 13.75) in chondrogenic group A and B, respectively. Like VAS and KOOS total at the one-year follow-up, the median Lysholm score in group B (76.50, IQR: 12.25) was significantly improved than group A (60, IQR: 30). At the two-year follow-up, the median Lysholm score was 62.50 (IQR: 35.5) and 73.50 (IQR: 18.25) in group A and B, respectively (Fig. 3c). Of importance, none of the two-year follow-up scores resulted in significantly different scores between the two chondrogenic groups. Both 65 cut-off of Lysholm score and MCID revealed that four donors from chondrogenic group A fell in the category of clinical failure along with one donor from group B. Remarkably, five donors from the low chondrogenic group (group B) were in the clinical success category (Fig. 3d). We did not find a significant correlation (*r* = −.308, *p* = 0.284) between in vitro chondrogenic potentials and clinical outcomes.

### Comparative expression of selected markers by the different chondrogenic and clinical outcome groups

**Fig. 4:**
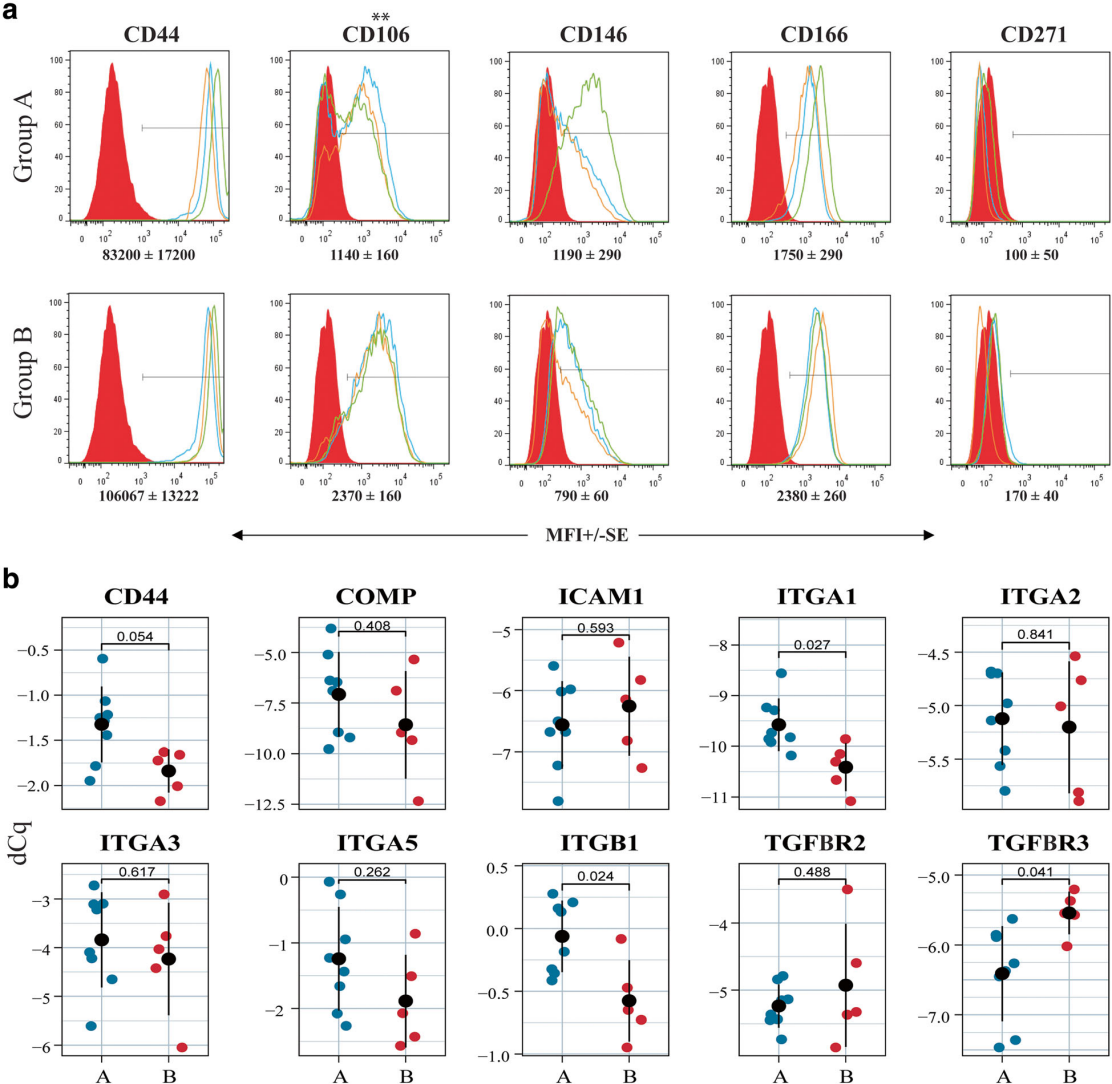
Comparison of selected molecular biomarkers between chondrogenic groups. **a** Surface protein expression of by flow cytometry from donors with high scores (*n =* 3; upper panels) and low scores (*n =* 3; low panels). Red peak represents the isotype control, and blue, orange and green peak represent expression by each independent donor. Average median fluorescence intensity (MFI) +/− standard error demonstrated differences in surface marker expression between two groups. **b** Analysis of selected genes of interest by qPCR revealed their relative expression in the high (*n =* 8) and low (*n =* 5) chondrogenic groups. Plotted values represent each donor, and the error bar represents standard deviation. Significance level, *p* (*) < 0.05

**Fig. 5:**
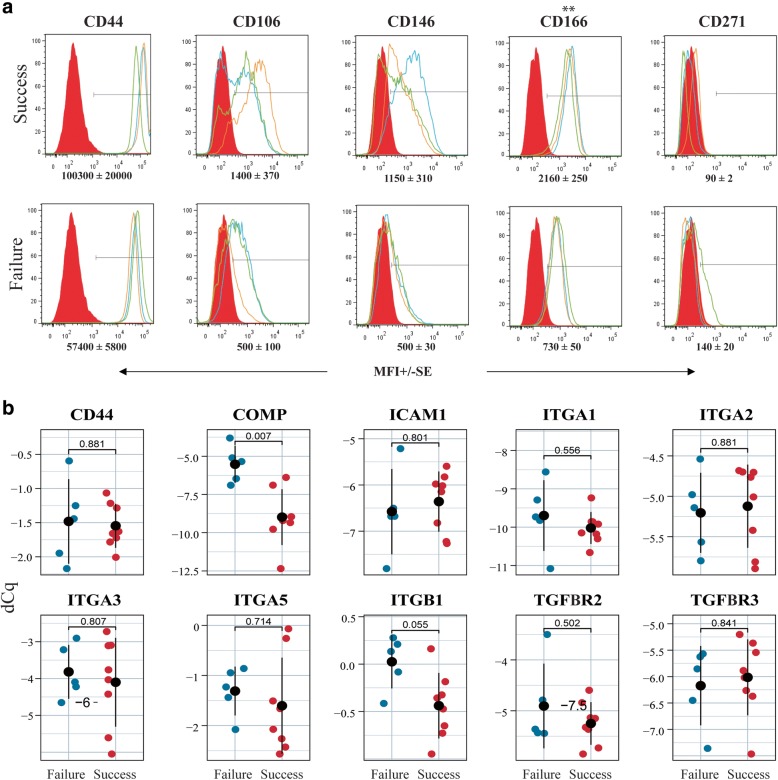
Comparison of selected molecular biomarkers between clinical groups. **a** Surface protein expression by flow cytometry from donors of clinical success (*n =* 3, upper panels) and failure (*n =* 3; low panels). Red peak represents the isotype control, and blue, orange and green peak represent the tested cell surface marker for each donor. Average median fluorescence intensity (MFI) +/− standard error demonstrated differences in surface marker expression between two groups. **b** Analysis of selected genes of interest by qPCR revealed their relative expression in the success (*n =* 8) and failure (*n =* 5) groups. Plotted values represent each donor, and the error bar represents standard deviation. Significance level, *p* (*) < 0.05 and (**) < 0.005

Flow cytometry was used to investigate putative surface markers of clinical outcomes and chondrogenicity (Figs. 4 and 5). We found a significantly higher expression of CD166 in the clinical success group compared to the failure group (MFI: 2160+/− 250 vs 730+/− 50) (Fig. 5a). The surface expression of CD44 was upregulated in the clinical success group in a near significant way (*p* = 0.054). Additionally, the expression of CD106 and CD146 was on average higher in the clinical success group compared to the clinical failure group (MFI: 1400+/− 370 vs 500+/− 100 and MFI: 1150+/− 310 vs 500+/− 30, respectively) (Fig. 5a), but the difference did not reach statistical significance.

When comparing the chondrogenic groups, the surface expression of CD106 (MFI: 2370+/− 160) was significantly higher in group B compared to group A (MFI: 1140+/− 160), thus suggesting a negative association with in vitro chondrogenic potential. We did not see significant differences in the surface expression of CD44 and CD166 between the two chondrogenic groups (Fig. 4a). The surface expression of CD146 was uneven among donors within the same chondrogenic group, and the expression was not indicative of chondrogenic potential (Fig. 4a). We also observed very low surface expression of CD271 in both chondrogenic and clinical groups (Figs. 4 and 5). Relative gene expression revealed significant upregulation of integrin-α1 (ITGA1) and integrin-β1 (ITGB1) in group A compared to group B, whereas TGF-β-receptor-3 (TGFBR3) expression was significantly downregulated in group A (Fig. 4b). In the clinical groups, the expression of cartilage oligomeric matrix protein (COMP) and ITGB1 were elevated in the failure group compared to the success group, but the expression of ITGB1 (*p* = 0.055) was barely significant (Fig. 5b). We did not detect significant differences in any of the studied genes associated with chondrogenic and clinical outcome categories (Additional file 2: Figure S2 and Additional file 3: Figure S3).

### An unbiased search of predictive biomarkers for in vitro chondrogenesis and ACI clinical outcomes by large-scale proteomics

**Fig. 6:**
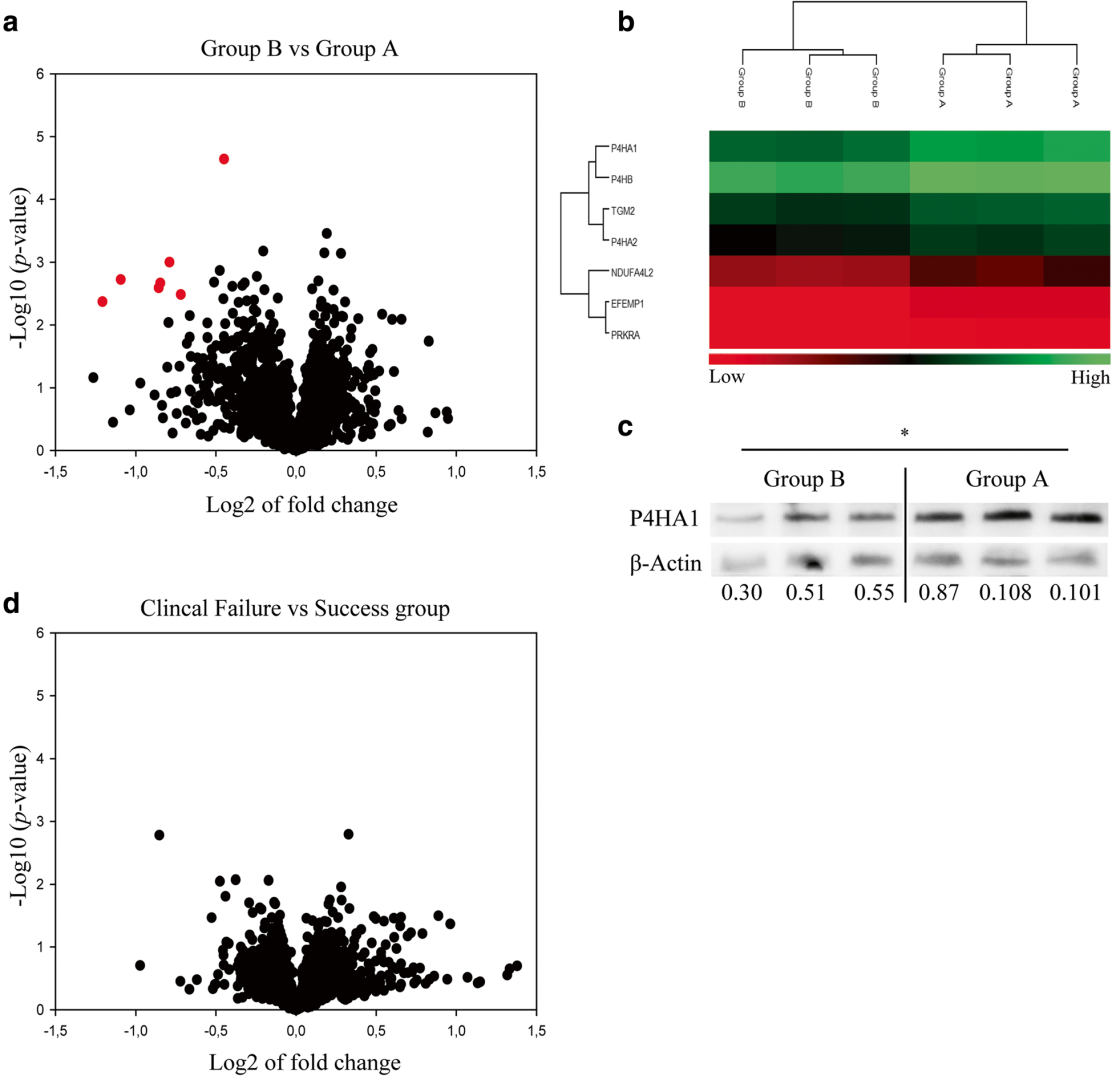
Comparative global protein expression analysis by LC-MS/MS between chondrocyte cultures associated with different chondrogenesis and clinical outcomes. **a** Volcano plot represents the expression of proteins in low chondrogenic samples (Group B) compared to high chondrogenic samples (Group A). Proteins underwent greater fold change, and lower *p*-value in the comparison are plotted further away from zero on X-axis and Y-axis, respectively. The red dots show significantly down-regulated proteins (FDR < 0.05) in chondrogenic group B. **b** Heat map showing the differentially expressed proteins when comparing chondrogenic groups. **c** Validation of P4HA1 protein expression by western blot. ImageJ software was used for relative quantification of P4HA1 (61 kDa) protein band to the loading control β-Actin (45 kDa). **d** Volcano plot represents the expression of proteins in clinical failure group compared to clinical success group. Significance level, *p* (*) < 0.05

A total of 2113 proteins were identified in cell extracts of chondrocytes from donors in the chondrogenic groups, of which 76 and 66 were classified as cell adhesion molecules and cell surface receptors, respectively, using the Kyoto Encyclopedia of Genes and Genomes (KEGG) database. In the cell extracts from chondrocytes of clinical success and failure groups, 2034 proteins were identified of which 74 and 59 were categorised as cell adhesion molecules and cell surface receptors, respectively. High throughput comparative analyses of identified proteins in the two chondrogenic groups revealed seven proteins significantly downregulated in group B compared to group A (Fig. 6a and b). Of relevance, P4HA1 (FDR < 0.01), an enzyme involved in collagen triple helix formation, was among the differentially expressed proteins. This outcome was validated in western blot analyses from all six donors (Fig. 6c). Moreover, we found no differentially expressed proteins when comparing donor cells belonging to the two clinical outcome groups (Fig. 6d).

## Discussion

The main objective of this study was to address the question if in vitro chondrogenic potential of donor-matched chondrocytes could predict clinical outcomes after ACI. We show that histological grading of pellets following chondrogenesis was not predictive of the clinical outcome. In an unbiased search for molecular markers of chondrogenesis, visual histological scoring of the 3D pellets was linked to the expression of the collagen synthesis enzyme, P4HA1.

It has been demonstrated that histological grading of pellets by Bern Score correlates significantly with biochemically assessed glycosaminoglycans content [30]. In line with other studies, we have also observed divergent in vitro chondrogenic potentials of culture-expanded chondrocytes from different donors [18, 20]. Due to unavoidable circumstances external to the experimental plan, the chondrocyte cultures included in this study were not synchronised at the same passage, but from passage 3–6 when preparing the pellets and RNA extracts for qPCR. In addition, other authors have proposed that the loss of cartilage phenotypic traits occur primarily during the first passages, and the cell phenotype becomes more stable after passage 3–4 [31]. We have confirmed by collagen type I/II gene expression that all donor chondrocytes were equally dedifferentiated at the start of 3D cultures (Fig. 2). In previous studies, we have shown that the chondrocytes redifferentiate in the 3D culture [25, 32]. Furthermore, neither patient’s age nor gender were associated with high or low in vitro chondrogenic potential (Table 1). Thus, we argue that the divergent donor-specific chondrogenic potential is at least to some degree donor-dependent and not associated to cell passage or donor demographics, which is in line with previous publications [18, 20].

The in vitro chondrogenic potential, as assessed by visual scoring of histological sections of 3D pellet cultures, was not associated with clinical success or failure two years after ACI. Earlier studies have proposed cell quality as one of the multiple parameters that may influence clinical outcomes after ACI [16, 22, 23]. In these studies, cell quality was evaluated by expression of cartilage-specific differentiation markers such as collagen type II and aggrecan, and other cell surface receptors such as FGFR3 and CD44. In a more recent study, the predictive clinical value of the suggested cell quality markers has been questioned [17]. In line with the later mentioned study, we did not observe correlations between the in vitro chondrogenic potency and clinical outcomes. There exist a number of possible explanations for our findings. After ex-vivo cell expansion, chondrocytes lose their phenotypic traits. It has been reported that the dedifferentiation of implanted chondrocytes impairs the quality of repaired tissue [33]. In addition, the process of cell redifferentiation is not comparable between in vitro and in vivo conditions. The fate of implanted chondrocytes after ACI and their contribution to rebuilding the damaged tissue is mostly unknown. Reports in pre-clinical models show varying proportions of implanted cells in the repaired tissue, but the majority of these studies indicate that most of the newly formed tissue is primarily composed of cells migrating to the lesion [34, 35]. Histologically, the repaired tissue after ACI appears predominantly fibro-cartilaginous [36]. However, it has been suggested that the quality of repair tissue from a histological point of view, does not always correlate with clinical outcomes [7, 10, 37]. Collectively, these observations and our results argue that the cell quality and the intrinsic chondrogenic capacity of implanted chondrocytes may not play a major role in the outcomes of the ACI procedure.

In previous studies aiming at identifying cell surface receptors that can predict chondrocytes with an improved chondrogenic potential in vitro, CD44, CD151 and CD146 have singled out as positively correlated with high chondrogenesis as judged by glycosaminoglycans content [20] or histological evaluation of spheroid cultures [38]. The CD44 protein expression has also been investigated in a clinical setting where a positive correlation between a clinical knee score at 24 months and CD44 protein expression in excess chondrocytes after ACI was found [22]. However, when Stenberg et al. analysed surplus chondrocytes from ACI, they found no correlation between CD44 gene expression and clinical outcome after three years [17]. We observed no differences in expression of CD44 when comparing chondrogenic groups. When looking at CD44 expression between the clinical groups, our findings are in line with Stenberg’s study, revealing no differences between the success and failure groups (Fig. 5).

We found elevated surface expression of vascular cell adhesion molecule 1 (CD106) in chondrocytes from donors displaying low chondrogenesis. A previous study reported the expression of CD106 in chondrocytes and their role as a marker for immunomodulation in inflamed joint [39]. However, in an early study from our group, comparing the chondrogenic potential of stromal cells from different tissue sources, we observed no association of CD106 surface expression with the chondrogenic potential of cells in vitro [25]. Hence, the role of CD106 in chondrogenesis may require further investigation. Importantly, we saw a significant upregulation of CD166 in the clinical success group. CD166 has been used as a marker to identify mesenchymal progenitor cells in cartilage [40, 41]. The expression of CD166 has been reported to be upregulated upon dedifferentiation [42], and others have observed expression changes also during redifferentiation [43]. However, there are no records of the predictive potential of CD166 in clinical outcomes. Our findings on CD166 represent an interesting lead with clinical relevance that deserves further investigation.

Several studies have implied that integrins, a group of cell surface receptors facilitating chondrocyte-matrix crosstalk, are central players in differentiation and chondrogenesis [20, 44]. Grogan et al. suggested ITGA3 as a marker for high chondrogenic potential, and also showed upregulation of ITGA5 and ITGA6 in chondrogenesis [20]. Another study investigating the effect of blocking ITGA1, ITGA5 and ITGB1 on chondrogenesis, reported early chondrogenesis was only inhibited by blocking ITGB1 [45]. Unlike their observations, we found ITGA1 and ITGB1 expression associated with high chondrogenesis, but no correlations of other integrin alpha units with chondrogenesis or clinical outcomes (Figs. 4 and 5). COMP, a matrix molecule, has previously been investigated as a potential biomarker. Unlike Wright et al. who found no correlation between COMP protein level in synovial fluid and clinical outcome [13], we found that the gene expression of COMP was significantly upregulated in the clinical failure group. Collectively, these observations suggest that markers associated with chondrogenesis of cells have limited or no value in clinical settings. Lastly, our gene expression analyses revealed significant upregulation of TGFBR3 gene in the poor chondrogenic group. We have not found any previous studies on TGFBR3 in relation to chondrogenesis. However, an upregulation upon dedifferentiation of chondrocytes has been suggested [46]. The clinical relevance of this finding is still uncertain.

The global proteomic approach to search for potential new biomarkers in cell-associated material revealed no differences between clinical success and failure group (Fig. 6). Similar observations were made by Stenberg et al. using global transcriptomics to compare clinical success and failure groups [17]. We found all subunits of the enzyme prolyl-4-hydroxylase among the seven proteins that were downregulated in the low chondrogenic group. Previous studies have reported gene and protein expression of P4HA1, P4HA2 and P4HB in human chondrocytes [47] and showed that they were induced by hypoxia. The role of P4HA1 in chondrogenesis is not yet defined, but given the critical role of this enzyme in the triple helix formation of newly formed collagens, our results suggest that P4HA1 (FDR < 0.01) could represent a promising biomarker to predict the cells with superior in vitro chondrogenic potential.

There are limitations of this study that need to be addressed. The relatively low number of patients included in the study may not give sufficient statistical power to find differences between the experimental groups. Hence the findings unveiled in the present study should be validated in larger cohorts. Additionally, in our chondrogenic assay, we have obviated the use of mechanical stimulation. Although being a dispensable factor to achieve redifferentiation of chondrocytes in vitro, the use of mechanical stimulation or longer in vitro culture has been shown to advance the maturation of neocartilage and entail superior redifferentiation potential [48, 49]. The use of a more advanced model incorporating mechanical stimulation could have produced a different result regarding the correlation between the in vitro chondrogenic potential and clinical outcome. Moreover, the clinical data represent short-term (two-year follow up) outcomes. A long-term follow-up in which the number of failures could increase might provide different scenarios [7]. We used Lysholm scores with a cut-off of 65 at two years postoperative to discern between clinical success and failure. However, we do not have records of factors that might have affected the healing process after ACI including lifestyle, impaired joint homeostasis, and concurrent use of medications. Finally, we do not have postoperative biopsies of the repair tissue so we are unable to make direct comparisons between the in vitro chondrogenic potential and the quality of the repaired tissue in vivo, which as mentioned earlier, may not necessarily have a direct correlation with clinical outcomes.

## Conclusions

This is the first study evaluating the in vitro chondrogenic potential of donor-matched chondrocytes and ACI clinical outcomes. The study shows that the cartilage-forming capacity of cells in vitro does not correlate with clinical outcomes for ACI. Additionally, the results reveal disparities between predictive markers of chondrogenesis and predictive markers of clinical outcomes. Furthermore, we provide insights into novel predictive biomarkers for chondrogenesis and clinical outcomes. The data presented in this study needs to be validated in a larger cohort of patients. However, our findings do not support the use of in vitro chondrogenic potential or molecular markers for chondrogenesis as predictive tools to be used in patient stratification for ACI.

## Additional files

Additional file 1: **Figure S1.** Chondrogenesis of culture-expanded chondrocytes in 3D pellets propagated in chondrogenic medium. Representative bright light microscopy images of histological sections (*n* = 14) and Bern scores. Proteoglycans stained with Alcian blue and the nuclei counterstained with Sirius red. (TIF 33702 kb)

Additional file 2: **Figure S2.** Comparison of selected genes between chondrogenic groups. Analysis of genes of interest by qPCR revealed their relative expression in the high (*n =* 8) and low (*n =* 5) chondrogenic groups. Plotted values represent each donor, and the error bar represents standard deviation. Significance level, *p* (*) < 0.05. (TIF 7762 kb)

Additional file 3: **Figure S3.** Comparison of selected genes between clinical groups. Analysis of selected genes of interest by qPCR revealed their relative expression in the success (*n =* 8) and failure (*n =* 5) clinical groups. Plotted values represent each donor, and the error bar represents standard deviation. Significance level, *p* (*) < 0.05. (TIF 7824 kb)

## Abbreviations

ACAN: Aggrecan
ACI: Autologous chondrocyte implantation
ACI-C: Autologous chondrocyte implantation-collagen membrane
AMIC: Autologous matrix-induced chondrogenesis
BMP-2: Bone morphogenic protein 2
COL2A1: Collagen type II α1
COMP: Cartilage oligomeric matrix protein
DMEM: Dulbecco’s Modified Eagle Medium
FBS: Foetal bovine serum
FGFR3: Fibroblast growth factor receptor 3
ITS: Insulin-transferrin-selenium
KEGG: Kyoto Encyclopedia of Genes and Genomes
KOOS: Knee injury and osteoarthritis outcome score
MACI: Matrix-assisted chondrocyte implantation
MCID: Minimal clinically important difference
MFI: Median fluorescence intensity
OA: Osteoarthritis
P4HA1: Prolyl-4-hydroxylase 1
RPL13A: Ribosomal protein L13a
TEAB: Triethylammonium bicarbonate
TGFBR3: TGF-β-receptor-3
TGF-β: Transforming growth factor β
VAS: Visual analogue scale
