# Supplementary figures and images for: In vitro chondrogenic potency of surplus chondrocytes from autologous transplantation procedures does not predict short-term clinical outcomes
Source: BMC Musculoskelet Disord. 2019 Jan 10;20:19. doi: 10.1186/s12891-018-2380-4 (PMC6329094; doi:10.1186/s12891-018-2380-4)

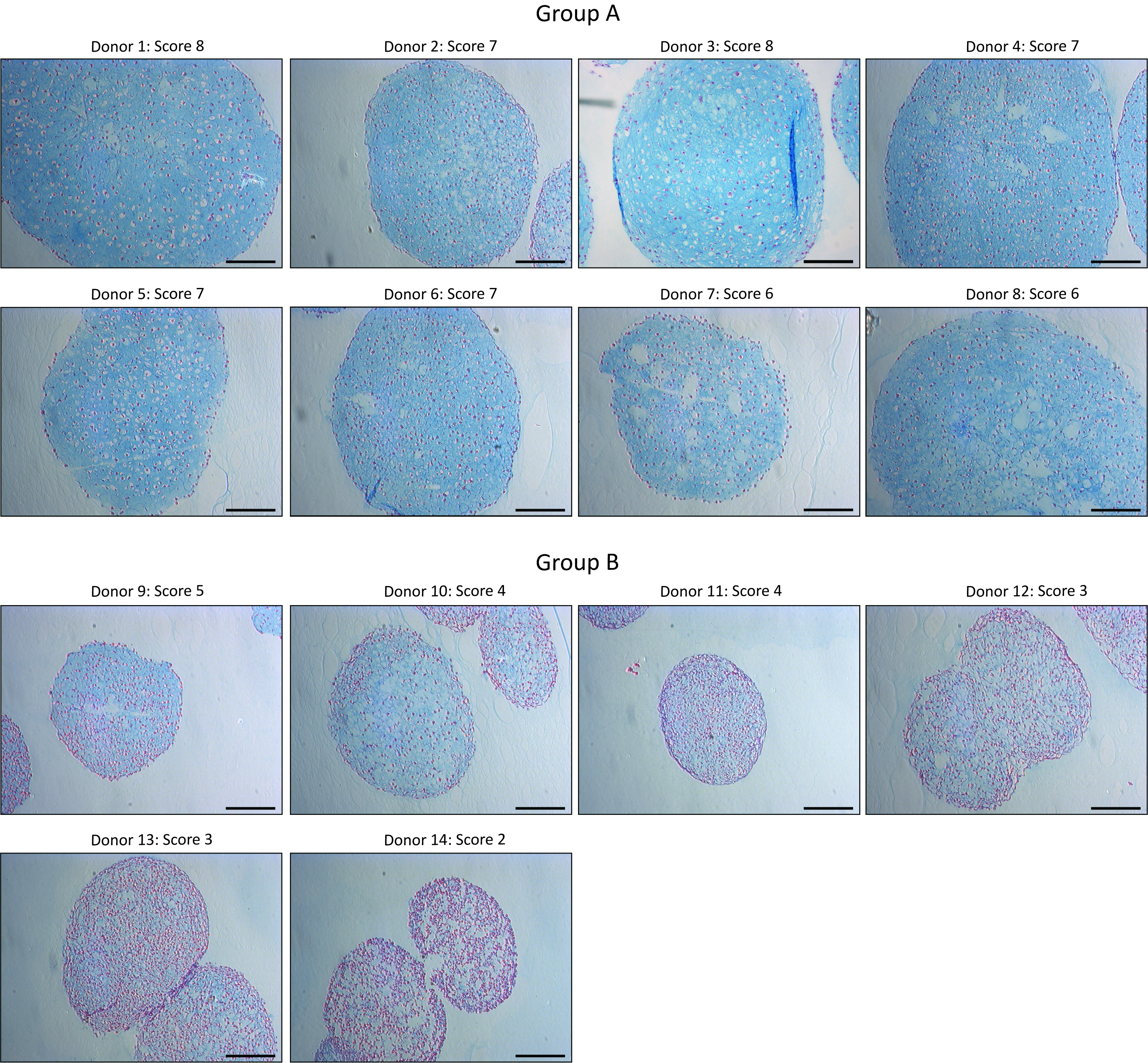

Supplement: Supplementary file 1 — Figure S1. Chondrogenesis of culture-expanded chondrocytes in 3D pellets propagated in chondrogenic medium. Representative bright light microscopy images of histological sections (n = 14) and Bern scores. Proteoglycans stained with Alcian blue and the nuclei counterstained with Sirius red. (TIF 33702 kb) [file 12891_2018_2380_MOESM1_ESM.tif]

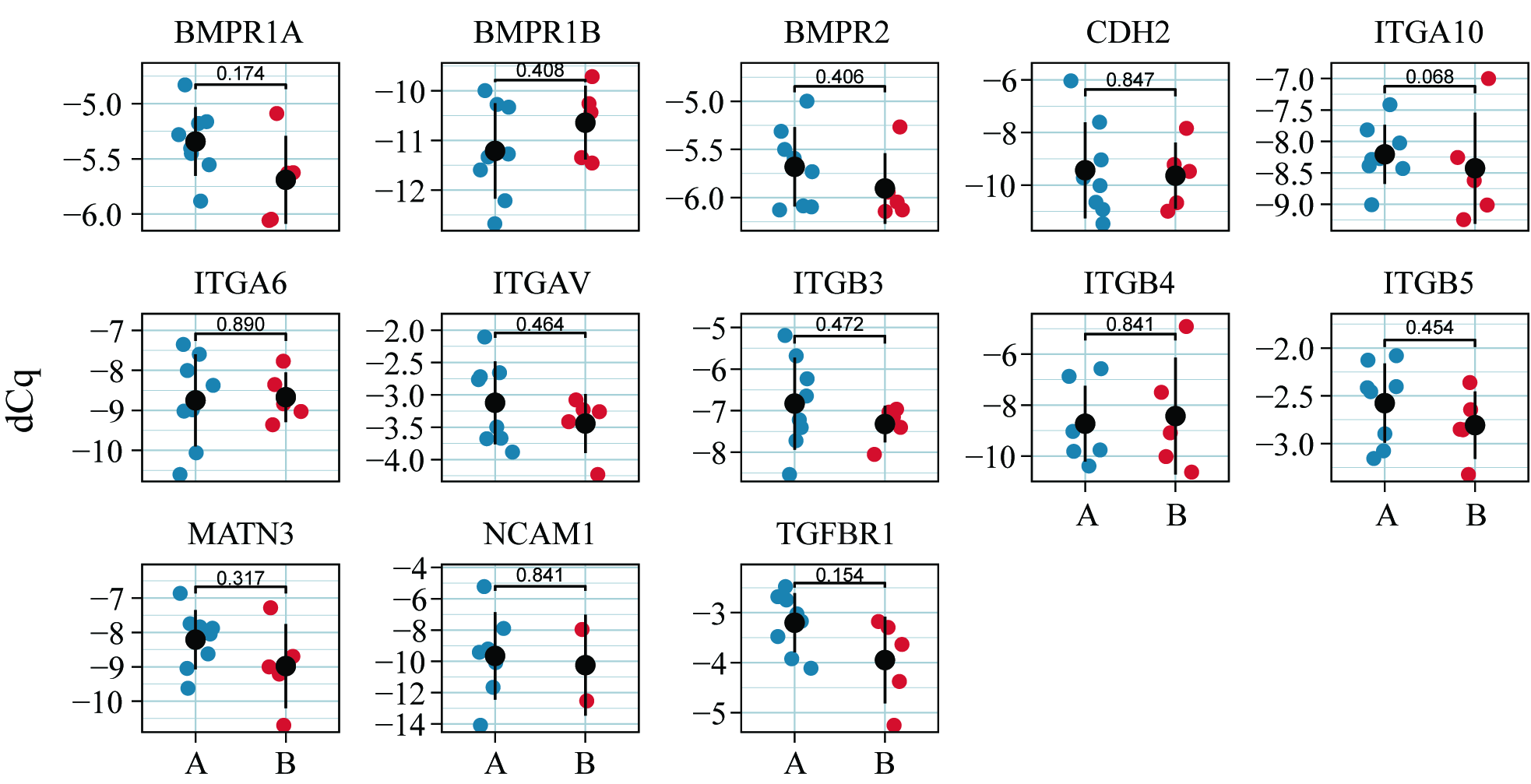

Supplement: Supplementary file 2 — Figure S2. Comparison of selected genes between chondrogenic groups. Analysis of genes of interest by qPCR revealed their relative expression in the high (n = 8) and low (n = 5) chondrogenic groups. Plotted values represent each donor, and the error bar represents standard deviation. Significance level, p (*) < 0.05. (TIF 7762 kb) [file 12891_2018_2380_MOESM2_ESM.tif]

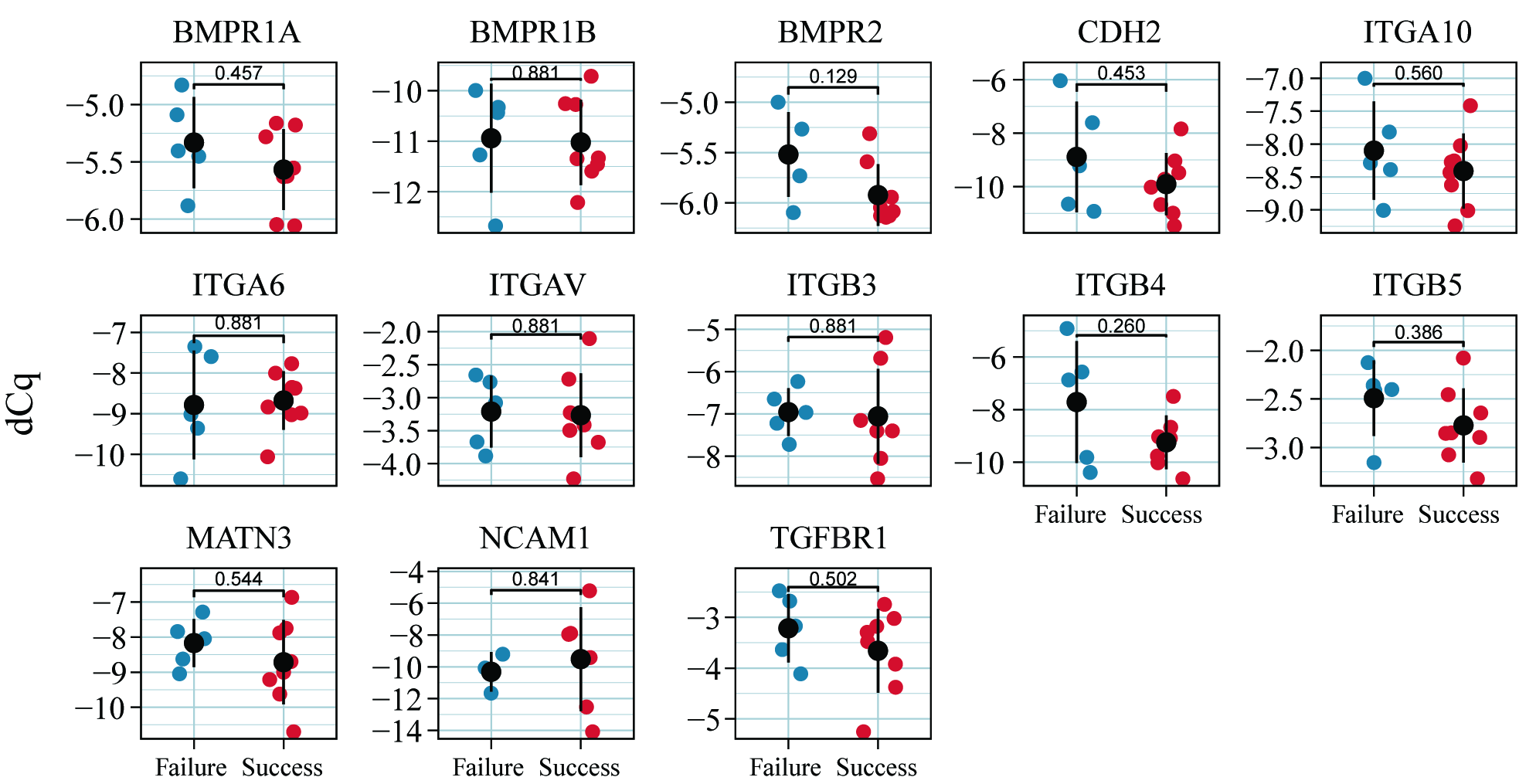

Supplement: Supplementary file 3 — Figure S3. Comparison of selected genes between clinical groups. Analysis of selected genes of interest by qPCR revealed their relative expression in the success (n = 8) and failure (n = 5) clinical groups. Plotted values represent each donor, and the error bar represents standard deviation. Significance level, p (*) < 0.05. (TIF 7824 kb) [file 12891_2018_2380_MOESM3_ESM.tif]
